# RAD-UNet: Research on an improved lung nodule semantic segmentation algorithm based on deep learning

**DOI:** 10.3389/fonc.2023.1084096

**Published:** 2023-03-23

**Authors:** Zezhi Wu, Xiaoshu Li, Jianhui Zuo

**Affiliations:** ^1^ Department of Computer Science, Anhui Medical University, Hefei, Anhui, China; ^2^ Department of Radiology, First Affiliated Hospital of Anhui Medical University, Hefei, Anhui, China; ^3^ Department of General Thoracic Surgery, The First Affiliated Hospital of Anhui Medical University, Hefei, Anhui, China

**Keywords:** deep learning, lung lesions, CT imaging, semantic segmentation, the U-Net, feature fusion, attention mechanism

## Abstract

**Objective:**

Due to the small proportion of target pixels in computed tomography (CT) images and the high similarity with the environment, convolutional neural network-based semantic segmentation models are difficult to develop by using deep learning. Extracting feature information often leads to under- or oversegmentation of lesions in CT images. In this paper, an improved convolutional neural network segmentation model known as RAD-UNet, which is based on the U-Net encoder-decoder architecture, is proposed and applied to lung nodular segmentation in CT images.

**Method:**

The proposed RAD-UNet segmentation model includes several improved components: the U-Net encoder is replaced by a ResNet residual network module; an atrous spatial pyramid pooling module is added after the U-Net encoder; and the U-Net decoder is improved by introducing a cross-fusion feature module with channel and spatial attention.

**Results:**

The segmentation model was applied to the LIDC dataset and a CT dataset collected by the Affiliated Hospital of Anhui Medical University. The experimental results show that compared with the existing SegNet [14] and U-Net [15] methods, the proposed model demonstrates better lung lesion segmentation performance. On the above two datasets, the mIoU reached 87.76% and 88.13%, and the F1-score reached 93.56% and 93.72%, respectively. Conclusion: The experimental results show that the improved RAD-UNet segmentation method achieves more accurate pixel-level segmentation in CT images of lung tumours and identifies lung nodules better than the SegNet [14] and U-Net [15] models. The problems of under- and oversegmentation that occur during segmentation are solved, effectively improving the image segmentation performance.

## 1 Introduction

Lung cancer has the highest mortality rate among cancers (1), and early diagnosis of lung cancer is very important for later treatment. Computed tomography (CT) (2) is a common imaging modality used to detect lung cancer and an important tool for early lung cancer diagnosis (3).

Computer-aided diagnosis (CAD) (4) systems can be applied to large numbers of CT images. Effective lesion information reduces misdiagnoses and missed diagnoses caused by manual reading, and CAD systems have become important tools in lung cancer diagnosis and treatment. Doctors use CAD systems to accurately locate and segment lung nodules and analyse the pathological characteristics of lung nodule lesions (5, 6). Since the segmentation results in lung nodule images directly affect pathological diagnoses, the accuracy of lung lesion segmentation algorithms is very important.

Traditional segmentation algorithms such as threshold segmentation (7), edge detection segmentation (8), and area growth (9, 10) can be used only in simple scenarios. For the segmentation of pulmonary lesions in medical images, due to the blurring of the surrounding grey region and the lack of differentiability with the background, traditional segmentation methods encounter several problems, such as missed and false edge detection.

The emergence of deep learning convolutional neural network (CNN) technology has further developed image segmentation methods and applied them in clinical practice. Image semantic segmentation plays an important role in the field of computer vision. The goal of image segmentation is to classify each pixel in an image, divide the image according to the specific, unique nature of different regions, and propose techniques and processes for identifying the target. In recent years, CNN and deep learning have been widely used in medical image analysis (11, 12), and FCN (13), SegNet (14) and U-Net (15) have demonstrated that convolutional neural networks can achieve good results not only in end-to-end learning but also in pixel-to-pixel learning.

The U-Net (15) image segmentation network (16) is a segmentation model with an encoder-decoder structure that has been widely used in image segmentation. In the network structure of U-Net (15), the left side includes an encoding structure, the right side includes a decoding structure, and the whole network has a U-shaped structure. U-Net’s encoder-decoder structure and jump connections have become a classic design, and several CNNs have been developed according to the core structure of U-Net (15). For example, Res-Unet (17) replaces each U-Net (15) submodule with a connection with a residual network module. The conditional random field (CRF) has been proposed to optimize the segmentation effect by using atrous convolutions to increase the receptive field while maintaining the resolution of the feature map. Atrous spatial pyramid pooling (ASPP) uses layers with different sampling rates to analyse a given input image in parallel, thereby capturing object features and image context information on a multidimensional scale. DeepLabv2 combines deep neural networks and probabilistic graph models to improve the localization of segmented target boundaries (18). Attention UNet (19) introduces the attention mechanism to U-Net (15), which combines the encoder features with the corresponding features in the decoder before proceeding to an attention module. Lin et al. proposed the feature pyramid network (FPN) (20) in 2017. The FPN model combines high- and low-resolution features and achieves an excellent image segmentation effect.

In 2022, Hong Huang et al. proposed domain-adaptive self-supervised transfer learning for chest CT classification of benign and malignant lung nodules and developed a data preprocessing strategy called adaptive slice selection to eliminate redundant noise in input samples with lung nodules (21). Ruoyu Wu et al. proposed a self-supervised transfer learning framework driven by visual attention (STLFVA) for benign and malignant recognition of nodules on chest CT Then, they used the multiview aggregate attention module to comprehensively recalibrate the multilayer feature map from multiple attention angles, which can strengthen the anti-interference ability of background information (22). Xu Shi et al. proposed a gastric cancer lesion detection network. A hierarchical feature aggregation structure is designed in the decoder, which can effectively fuse deep and shallow features. The attention feature fusion module is introduced to accurately locate the lesion area, and the attention features of different scales are fused to obtain rich lesion discrimination information (23).

In hepatoma cell nuclear segmentation, Shyam et al. (24) designed a NucleiSegNet that includes a residual block, a bottleneck block, and an attention module. Anirudh et al. (25) proposed an encoder-decoder network combining the atrous spatial pyramid pool and attention module for renal cell nuclei segmentation. Massimo et al. (26) adopted a hybrid segmentation strategy based on gland contour structure and deep learning in prostate cancer detection. In breast cancer HI segmentation, David et al. (27) proposed a deep multiploid network to extract spatial features within classes and learn spatial relationships between classes. Blanca et al. (28) designed an encoder-decoder network that combines separable void convolution and conditional random fields. Amit et al. (29) introduced a separable convolutional pyramid pooling network and achieved good performance on renal and breast HIs.

## 2 Problems and scenarios

Lung lesion image segmentation and typical image segmentation have some important differences. Lung image lesion segmentation targets tend to be small; thus, the proportion of lesion pixels in the image is small, and small target features are difficult to identify. Moreover, convolutional neural network training is more difficult. Furthermore, the similarity between lung lesions and the imaging environment is very high, and highly recognizable features are difficult to extract. Traditional image segmentation networks are less effective for segmenting small targets that cannot be clearly distinguished, such as lung image lesions with similar image backgrounds. Based on the above lung image segmentation difficulties, the U-Net (15) segmentation algorithm is improved.

First, the encoder in the U-Net (15) model was improved. The U-Net (15) encoder was improved by introducing a series of ResNet neural networks with residual structures, and multiple ResNet models are used as encoders to verify the segmentation effect in the experiment to improve the segmentation performance of the proposed network.

Second, an atrous spatial pyramid pooling (ASPP) module is added after the U-Net (15) encoding structure. Based on the characteristics of lung lesions, which are smaller segmentation targets, the ASPP module samples the given input in parallel with different convolutional layers to capture the image context at multiple scales. This multiscale information is integrated to enhance the feature extraction ability of the proposed model.

Third, a dual feature cross-fusion (DFCF) module is proposed. The introduction of the attention mechanism allows the convolutional neural network to focus on more important features in the image, thereby reducing the attention to unimportant features and targeting the lesions in lung images. Considering the high environmental similarity, the DFCF module uses a channel attention mechanism to cross-integrate global and local semantic features, thereby enhancing the ability of the model to extract highly recognizable features. Moreover, the channel attention mechanism in the DFCF module is improved to a convolutional block attention module (CBAM) with both channel and spatial attention, thus allowing the proposed model to consider different locations in the same channel at the same time. The importance of the different channel pixels enhances the performance of the proposed network model. Taking U-Net (15) as the backbone network, an encoder-decoder network model known as RAD-UNet with ResNet residual structure, ASPP and DFCF modules is proposed.

## 3 Structure and improvement

### 3.1 U-Net network structure

**Figure 1:**
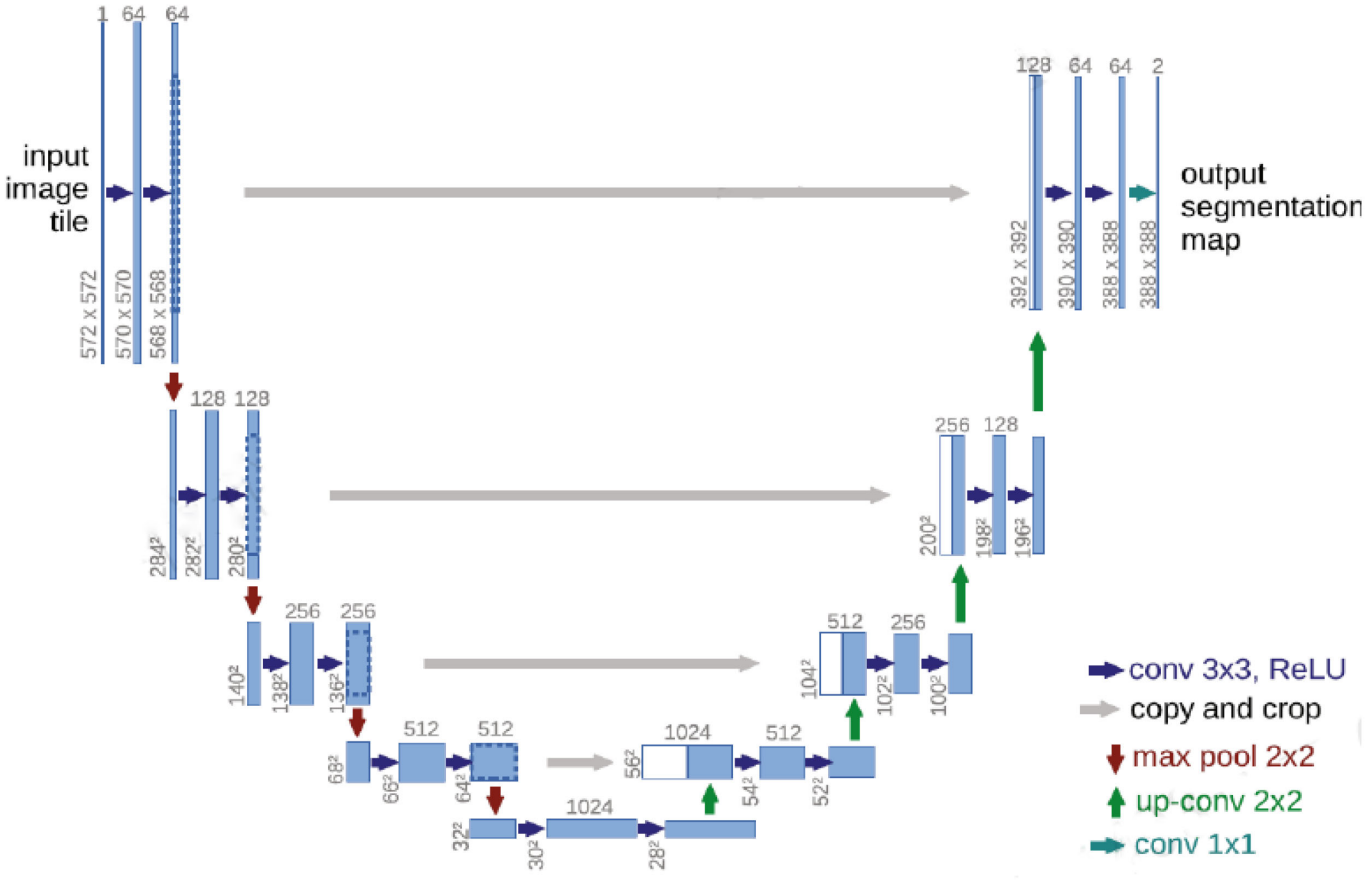
Schematic diagram of the U-Net (15) network structure.

In 2015, Ronneberger et al. proposed a U-Net (15) image segmentation network with an encoder-decoder structure. The U-Net (15) model has a U-shaped symmetrical structure, which is shown in **Figure 1**.

The left side of the U-Net (15) convolutional neural network includes the convolutional and pooling layers, and the right side includes an upsampling layer. Each convolutional layer in U-Net (15) obtains a feature map, which is transmitted to the corresponding upsampling layer through jump connections, thus ensuring that the feature map of each layer is involved in subsequent calculations.

The U-Net (15) convolutional neural network effectively utilizes the features in the low-level feature map to ensure that the final feature map contains both high-level features and low-level features, thereby realizing the fusion of the extracted features at different scales and improving the accuracy of the U-Net (15) model.

The left half of U-Net (15) includes five downsampling modules, which each consist of two 3×3 convolutional layers, the ReLU activation function, and a 2×2 maximum pooling layer. The right half of U-Net (15) includes four upsampling modules, which each consist of an upsampling convolutional layer, feature stitching, two 3×3 convolutional layers, and the ReLU activation function. U-Net (15) fuses features through feature map stitching, thus obtaining a network with richer features.

### 3.2 RAD-UNet network improvement model

**Figure 2:**
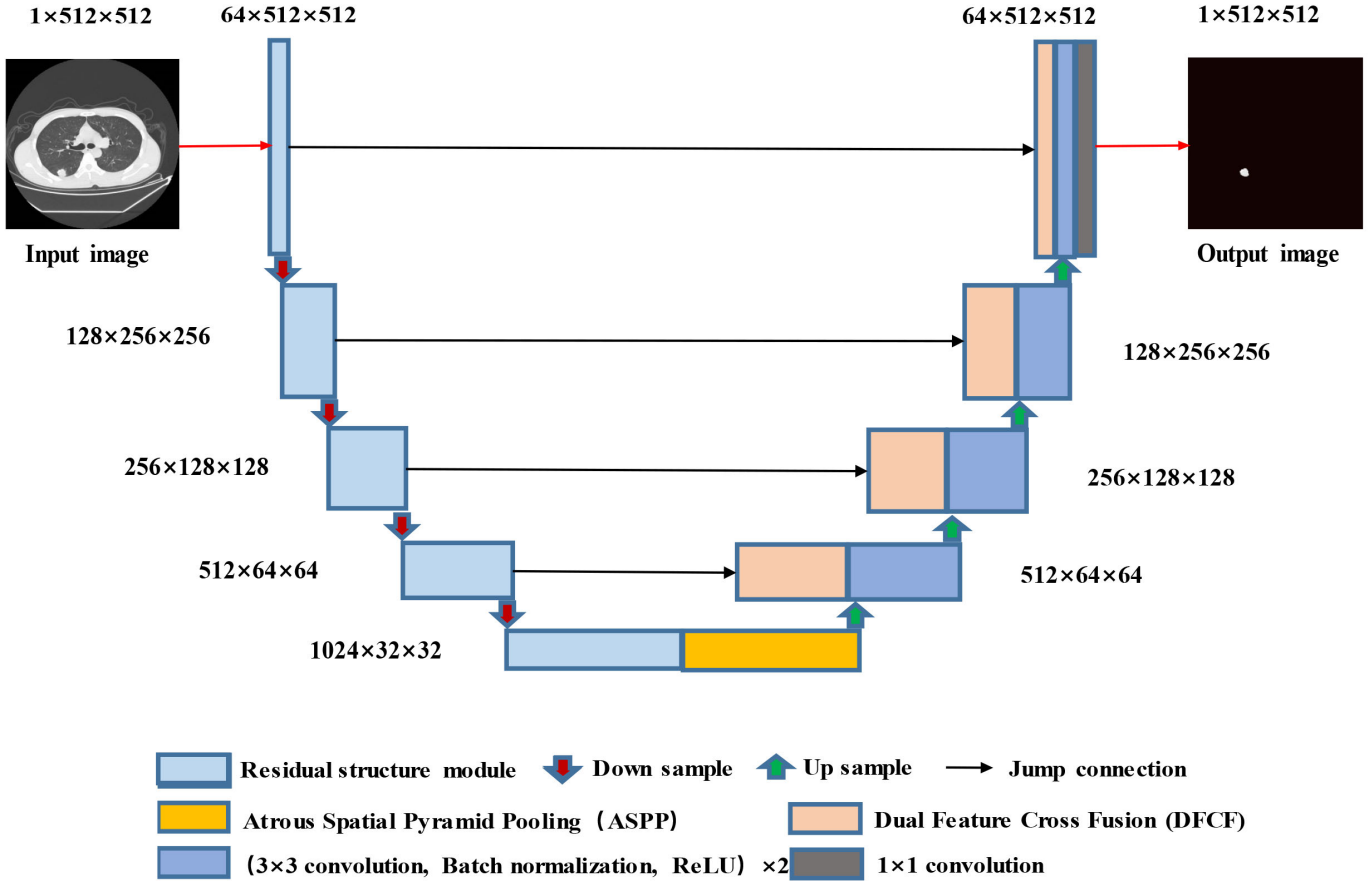
Structure diagram of the RAD-UNet convolutional neural network.

The proposed RAD-UNet model is based on an improved U-Net (15) segmentation model, and the improved cavity convolution enhances the lung lesions in the input images. The ability of the network to extract smaller target features, the expansion of the receptive field, and the fusion of the feature maps extracted by different layers improve the lung image segmentation effect for smaller lesions. Furthermore, the improved convolution in RAD-UNet is enhanced by using a nonlocal attention mechanism, which combines local and global important semantic features at different levels, thereby improving the ability of the network to distinguish between lung lesions and the image background. The proposed model uses U-Net (15) as the backbone network, and the structure of the proposed RAD-UNet is shown in **Figure 2**.

The RAD-UNet convolutional neural network model shown in **Figure 2** is based on an improved U-Net (15) model. First, the encoder in U-Net (15) is improved by incorporating network modules with residual structures. In addition, an ASPP module is added after the U-Net (15) encoder, which is shown in yellow in **Figure 2**. As shown in the figure, DFCF modules are added after each upsampling layer in the decoder.

When a lung lesion image is input into the network, after each downsampling layer, the input enters the improved ASPP module, which performs multiscale feature fusion. The output is obtained after the upsampling layers, and four DFCF modules are introduced in the upsampling process. The DFCF modules integrate important features at different levels, improving RAD-UNet’s lung lesion segmentation performance and the ability to distinguish between the target and the background.

#### 3.2.1 RAD-UNet encoder improvements

**Figure 3:**
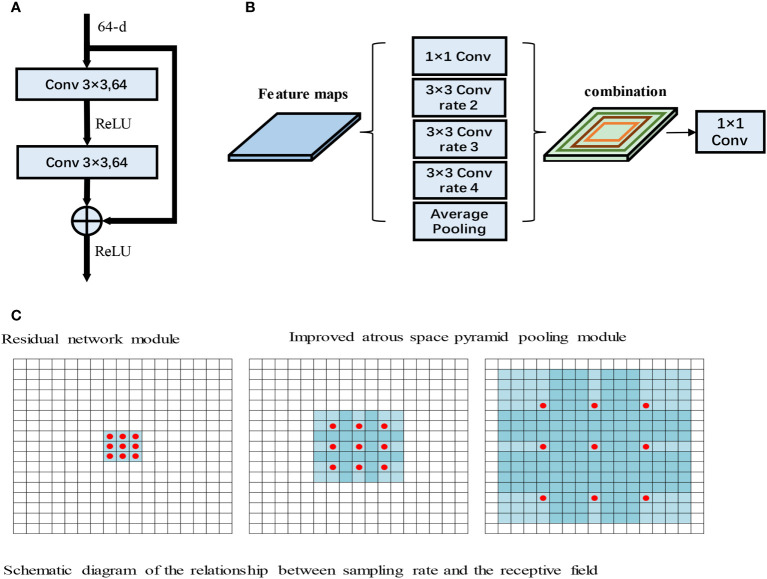
**(A)** Residual network module. **(B)** Improved atrous space pyramid pooling module. **(C)** Schematic diagram of the relationship between the sampling rate and the receptive field (31).

Due to the gradual increase in the number of layers, convolutional neural network models often encounter gradient explosion and network degradation (30). To solve these issues, He et al. proposed the deep residual network (ResNet) model. ResNet is a neural network model that includes several stacked residual blocks, and the residual block structure is shown in **Figure 3A**.

As shown in **Figure 3A**, the input to the residual module is output after passing through two 3×3 convolutions. The features then pass through the ReLU activation function to obtain the final output. ResNet has powerful characterization capabilities and enhances performance in image segmentation applications. The RAD-UNet network model proposed in this paper replaces the U-Net (15) encoder with ResNet residual blocks, and the decoder uses the original U-Net (15) decoding structure. In the improved RAD-UNet model, the increase in the number of neural network layers allows stronger and richer features to be obtained, thereby improving the segmentation effect.

#### 3.2.2 RAD-UNet ASPP module

Convolutional neural networks often need to introduce more convolutional layers and pooling layers, which decreases the resolution of the feature map and increases the computational complexity of the model. Atrous convolutions (31) can arbitrarily enlarge the receptive field without introducing additional parameters; thus, atrous convolutions do not reduce the resolution of the feature map. Atrous convolutions use the dilated rate parameter to enlarge the receptive field, thus allowing the convolutional layer to have a larger receptive field without downsampling the same number of parameters or performing the same number of computations. For example, for a 3×3 convolution, when the sampling rate is 1, the receptive field is 3×3; however, when the sampling rate is 2, the receptive field is 7×7, and when the sampling rate increases to 4, the receptive field increases to 15×15. The relationship between the sampling rate and the receptive field is shown in **Figure 3B**.

In **Figure 3B**, the red dots represent the convolutional nuclei in the hollow convolutions, and the blue grids represent the size of the receptive field. The introduction of atrous convolutions increases the receptive field, thereby allowing important multiscale information to be obtained while preventing information loss.

Chen et al. introduced the ASPP module into the DeepLab neural network. The ASPP module has achieved good results in extracting multiscale image features. The smaller the sampling rate of the ASPP module, the better the module segments smaller targets, and the larger the sampling rate, the better the module segments larger targets.

In this paper, the ASPP module is added after the U-Net (15) encoding structure, and the ASPP module improves the ability of the model to identify small lung lesions in the input images. Moreover, the ASPP module enhances the ability to extract the characteristics of small targets and uses different convolution layers to fuse multiscale information, thereby enhancing the feature extraction ability of the model. The improved ASPP module is shown in **Figure 3C**.

**Figure 3C** shows the improved ASPP module in RAD-UNet. The sampling rate of the ASPP module is 2, 3, or 4. The improved ASPP module consists of a 1×1 convolution, 3×3 convolutions with sampling rates of 2, 3 or 4, and an average pooling layer. Convolutions with sampling rates of 2, 3 and 4 increase the ability of the neural network to segment smaller targets, such as lung lesions, thus improving the model segmentation effect.

#### 3.2.3 RAD-UNet DFCF module

The attention mechanism in deep learning allocates computing power to more important information and filters secondary information to retain important information, similar to the attention mechanism in human vision. In deep learning convolutional neural networks, the attention mechanism considers the distribution of network weights, and in computer vision tasks such as semantic segmentation, the attention mechanism is focused on learning the area of interest in the image.

**Figure 4:**
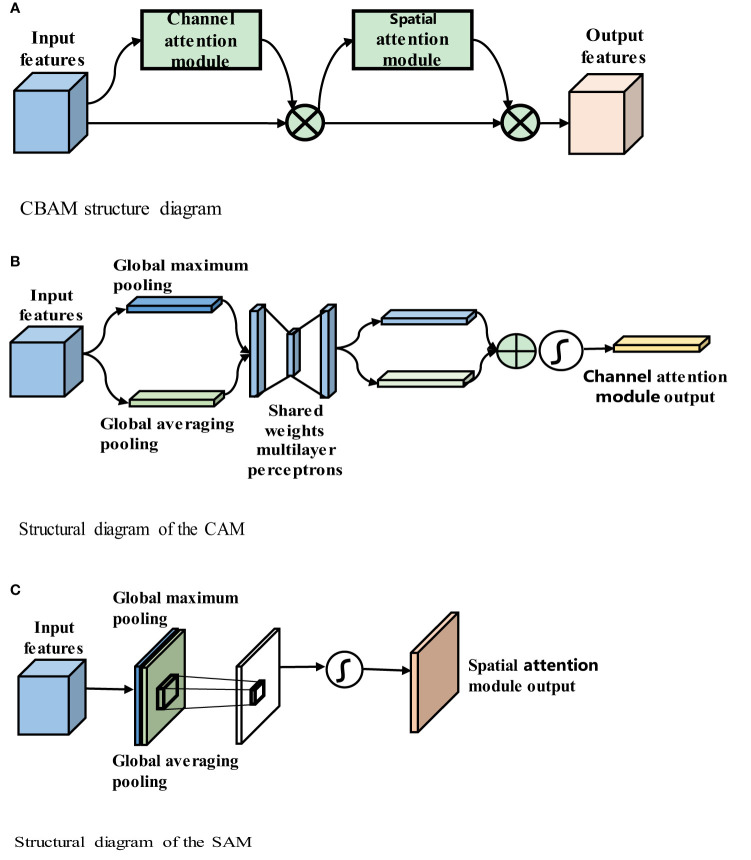
**(A)** CBAM structure diagram. **(B)** Structural diagram of the CAM. **(C)** Structural diagram of the SAM.

Convolutional neural networks perform well in image analysis and processing tasks (e.g., image segmentation and object detection (32)). In a convolutional neural network, the hierarchical pattern in the global receptive field is captured by inserting nonlinear activation functions and downsampling convolutional layers. To obtain better training results in convolutional neural networks without introducing excessive computations, the convolutional block attention module (CBAM) (33), which combines spatial and channel attention in two dimensions, is introduced. The CBAM is a lightweight attention module that was proposed by Woo et al., and its structure is shown in **Figure 4A**.

**Figure 4A** shows that the CBAM contains a channel attention module (CAM) and a spatial attention module (SAM). The CBAM uses both channel and spatial attention to calculate the attention feature map, which is then multiplied by the input feature map to improve the network focus and produce more reliable features. The structures of the CAM and SAM are shown in **Figures 4B, C**, respectively.

**Figure 4B** shows that input feature F is H × W × C, where H denotes the height of the feature map, W denotes the width, and C denotes the number of channels, global average pooling and global maximum pooling are used to obtain two 1 × 1 × C feature maps, and these two feature maps are fed into the two-layer fully connected neural network with shared parameters. The two feature maps obtained are added, and the weight coefficient between 0 and 1 is obtained by the sigmoid function. Then, the weight coefficient is multiplied by the input feature map to obtain the final output feature map, as shown in (3-1)


(3 1) $$M_{c}\left(F\right)=\;\sigma \left(MLP\left(AvgPool\left(F\right)\right)+MLP\left(MaxPool\left(F\right)\right)\right)=\sigma (W_{1}(W_{0}())+W_{1}(W_{0}()))$$

In the above equation, MLP (Multilayer Perceptron) represents the shared MLP module in the channel attention module,where σ denotes the sigmoid function, W_0_∈R^C/r×C^, and W_1_∈R^C×C/r^. The MLP weights, W_0_ and W_1_, are shared for both inputs, and the ReLU activation function is followed by W_0_. .. and $F_{\max}^{c}$ denote average-pooled features and max-pooled features respectively.

**Figure 4C** shows that the input feature F is H x W x C. The maximum pooling and average pooling of one channel dimension are carried out to obtain two H x W x 1 feature maps. These two feature maps are spliced together in the channel dimension as H x W x 2 after a convolutional layer. Reduced to 1 channel, the convolution kernel uses 7 × 7, while keeping H and W unchanged. The output feature map is H x W x 1, and then the spatial weight coefficient is generated by the sigmoid function. The final feature map is obtained by multiplying with the input feature map, as shown in (3-2):


(3-2) $$M_{s}\left(F\right)=\;\sigma \left(f^{7x7}\left(\left[AvgPool\left(F\right)\right);\;MaxPool\left(F\right)\right]\right))=\sigma (f^{7x7}([F_{avg}^{s};F_{\max}^{s}]))$$

where σ denotes the sigmoid function, and f^7x7^ represents a convolution operation with a filter size of 7 × 7. $F_{avg}^{s}\in R^{1}x\;H\;x\;Wand\in F_{\max}^{s}R^{1}x\;H\;x\;W$ The CBAM includes both channel and spatial attention mechanisms and fewer parameters and obtains important feature information through learning. The CBAM’s channel attention module, CAM, uses parallel global maximum pooling and global averaging pooling to extract richer and more comprehensive high-level feature information. The results obtained by the global maximum pooling and global average pooling layers in the CAM are added, and the sigmoid activation function is used to obtain the CAM output. The CBAM concatenates the spatial attention module (SAM) after the channel attention module. The SAM performs global maximum pooling and global average pooling on the input features and then combines and consoles the two features. The new feature map is then passed through a sigmoid activation function, and the output is multiplied by the original input to obtain the final result.

#### 3.2.4 The algorithm in the improved DFCF module

**Figure 5:**
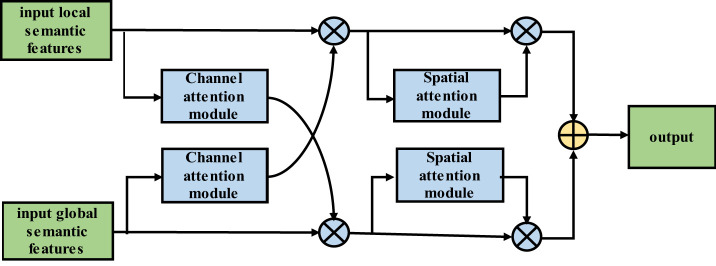
Improved two-feature cross-fusion structure diagram.

The input local semantic features (LSFs) pass through the channel attention module, which includes global average pooling and global maximum pooling layers; then, the features pass through a fully connected layer, the ReLU activation function, and another fully connected layer. Next, the features are added and passed through the sigmoid activation function. Finally, the result is multiplied by the global semantic features (GSFs) to obtain the final output of the channel attention module. The output then passes through the SAM, that is, the GSFs first pass through global average pooling and global maximum pooling layers. Then, the results are combined and passed through a convolutional layer. Next, the results pass through a sigmoid activation function. The resulting output is multiplied by the input to obtain the final output of the SAM. The structure of the improved DFCF module is shown in **Figure 5**.

Similar to the LSF algorithm flow, the input GSFs pass through the CAM, that is, the features pass through global average pooling and global maximum pooling layers. Then, the features pass through a fully connected layer, the ReLU activation function, and another fully connected layer before they are combined. The result is passed through the sigmoid activation function, and the output is multiplied by the LSFs to obtain the output of the CAM. The output then passes through the SAM; that is, the LSFs first pass through global average pooling and global maximum pooling layers. The results are then combined and passed through a convolutional layer, and the output is passed through the sigmoid activation function. The resulting output is then multiplied by the input to obtain the final output of the SAM.

The improved DFCF module takes into account not only the importance of the pixels in different channels but also the importance of the pixels at different locations in the same channel.

#### 3.2.5 Loss function

Due to the small proportion of lung lesion pixels in the CT image, the samples are imbalanced in the dataset. When the model is optimized using the binary cross entropy loss (BCE loss) function, the segmentation accuracy of small lesions is not high. The BCE loss function formula is shown in Equation (3.3).


(3.3) $$L_{BCE}(y,ŷ)=-(y\log(ŷ)+(1-y)\log(1-ŷ))$$

The Dice loss function can alleviate the sample imbalance in lung image datasets; however, the Dice loss function may produce gradient oscillations during training and is not as stable as the BCE loss function. The Dice loss function formula is shown in Equation (3.4).


(3.4) $$L_{DICE}(y,ŷ)=1-(2yŷ+1)/(y+ŷ)$$

This paper adopts a loss function L that combines the Dice and BCE loss functions, and its formula is shown in Equation (3.5):


(3.5) $$L(y,ŷ)=L_{BCE}(y,ŷ)+L_{DICE}(y,ŷ)$$

In the above formula, y represents the actual label value, and y represents the model prediction result.

## 4 Experimental data and evaluation indicators

The experimental datasets used in this paper are the public Lung Image Database Consortium (LIDC) dataset and the pulmonary CT dataset of the Affiliated Hospital of Anhui Medical University (AHAMU-LC). The LIDC dataset was collected by the National Cancer Institute and includes a total of 1018 cases. In each case, four radiologists identified the contours of the lung nodules and other signs of disease. The AHAMU-LC dataset includes a total of 436 cases, and the CT images in each case were labelled and validated by multiple imaging physicians. The dataset includes 9265 slices with lung nodules, and the captured images are 512 ×512 in size.

In this paper, evaluation indicators based on a confusion matrix are adopted. The mean of the intersection over union (mIoU), recall, precision and F1-score (34, 35) are adopted as indicators to evaluate the performance of the proposed network.

**Figure 6:**
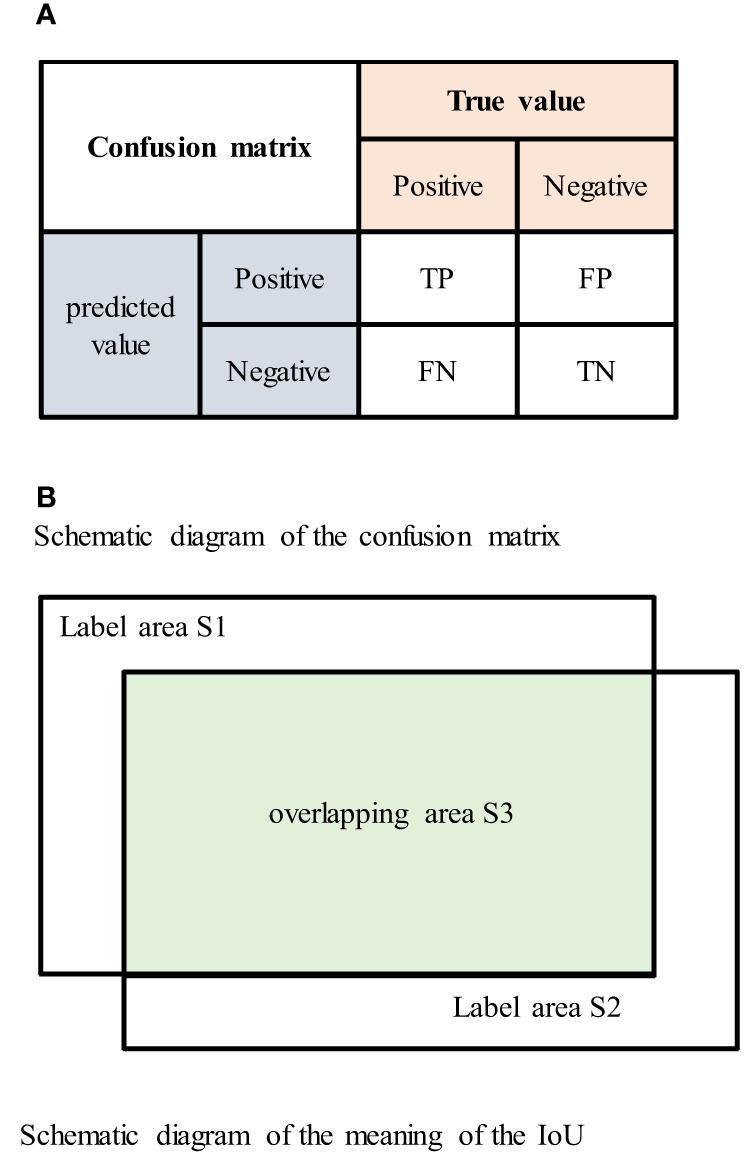
**(A)** Schematic diagram of the confusion matrix. **(B)** Schematic diagram of the meaning of the IoU.

In the confusion matrix, positive and negative represent positive and negative samples, respectively. True positive (TP) indicates that the true class of the sample is a positive example, and the model prediction result is also a positive example; true negative (TN) indicates that the true class of the sample is negative, and the model prediction result is also negative; false positive (FP) indicates that the true class of the sample is negative but the model prediction result is positive; and false negative (FN) indicates that the true class of the sample is positive but the model prediction result is negative. The confusion matrix is shown in **Figure 6A**.

The intersection over union (IoU) is the ratio of the intersection and union of the true labels and segmentation results. The IoU value ranges between 0 and 1, and larger IoU values indicate better segmentation results. The IoU is represented by the area in **Figure 6B** as $IoU=\frac{S_{3}}{S_{1}+S_{2}-S_{3}}$ The formula for the IoU is shown in Equation (4.1) (29), the formula for the confusion matrix is shown in Equation (4.2) (30), and the mIoU formula is shown in Equation (4.3).


(4.1) $$IoU=\frac{|A_{P}\cap A_{GT|}}{|A_{P}\cup A_{GT|}}$$

(4.2) $$IoU=\frac{TP}{TP+FP+FN}$$

(4.3) $$mIoU=\frac{1}{n}\sum_{i-1}^{n}IoU_{i}$$

The recall rate represents the proportion of positive pixels that were correctly identified divided by the total number of positive pixels. The recall formula is shown in Equation (4.4) (36), and the formula for the confusion matrix is shown in Equation (4.5) (37).


(4.4) Recall=|AP∩AGT||AGT|

(4.5) Recall=TPTP+FN

The precision indicates the proportion of pixels that were correctly segmented to the total number of segmented pixels. The precision formula is shown in Equation (4.6) (36), and the formula for the confusion matrix is shown in Equation (4.7) (37).


(4.6) $$\mathrm{Pr}ecision=\frac{|A_{P}\cap A_{GT}|}{|A_{P}|}$$

(4.7) $$\mathrm{Pr}ecision=\frac{TP}{TP+FP}$$

The F1-score represents the harmonized average of the precision and recall. The F1-score varies between 0 and 1, and the formula is shown in Equation (4.8) (36).


(4.8) F1−score=2xRecallxPrecisionRecall+Precision

In the above formula, | A_p_ | represents the area of the human body in which the network segments the lung lesions in the CT images, and |A_GT_| represents the actual area of the CT lung image lesion label. The index value is in the test set of all lung imaging lesions and is calculated by taking the average of the calculation results.

## 5 Experimental results and analysis

### 5.1 Experimental environment and experimental settings

In medical imaging, since various tissue structures and lesions often have different CT values, the range of CT values of interest is typically selected using window position and window width techniques. For lung nodule segmentation in CT images, the window width of the LIDC and AHAMU-LC datasets was optimally set. For the LIDC dataset, a window position of 250 and window width of 1490 were selected (38). For the AHAMU-LC dataset, a window position of 500 and window width of 1490 were selected. Moreover, the data domain of the CT image was normalized to the range of [0, 1].

A total of 9657 lung CT lesions were taken from the LIDC and AHAMU-LC datasets, including images with corresponding manual segmentation results. The data were randomly divided into training and test sets at a ratio of 8:2. The data were preprocessed using left-right and up–down flips for CT image enhancement, which improves the generalizability of the model and reduces overfitting. During training, the initial learning rate was set to 0.001, the batch size was set to 64, the number of epochs was set to 300, and the model was trained using the Adam optimization algorithm.

### 5.2 LIDC experimental results

**Figure 7:**
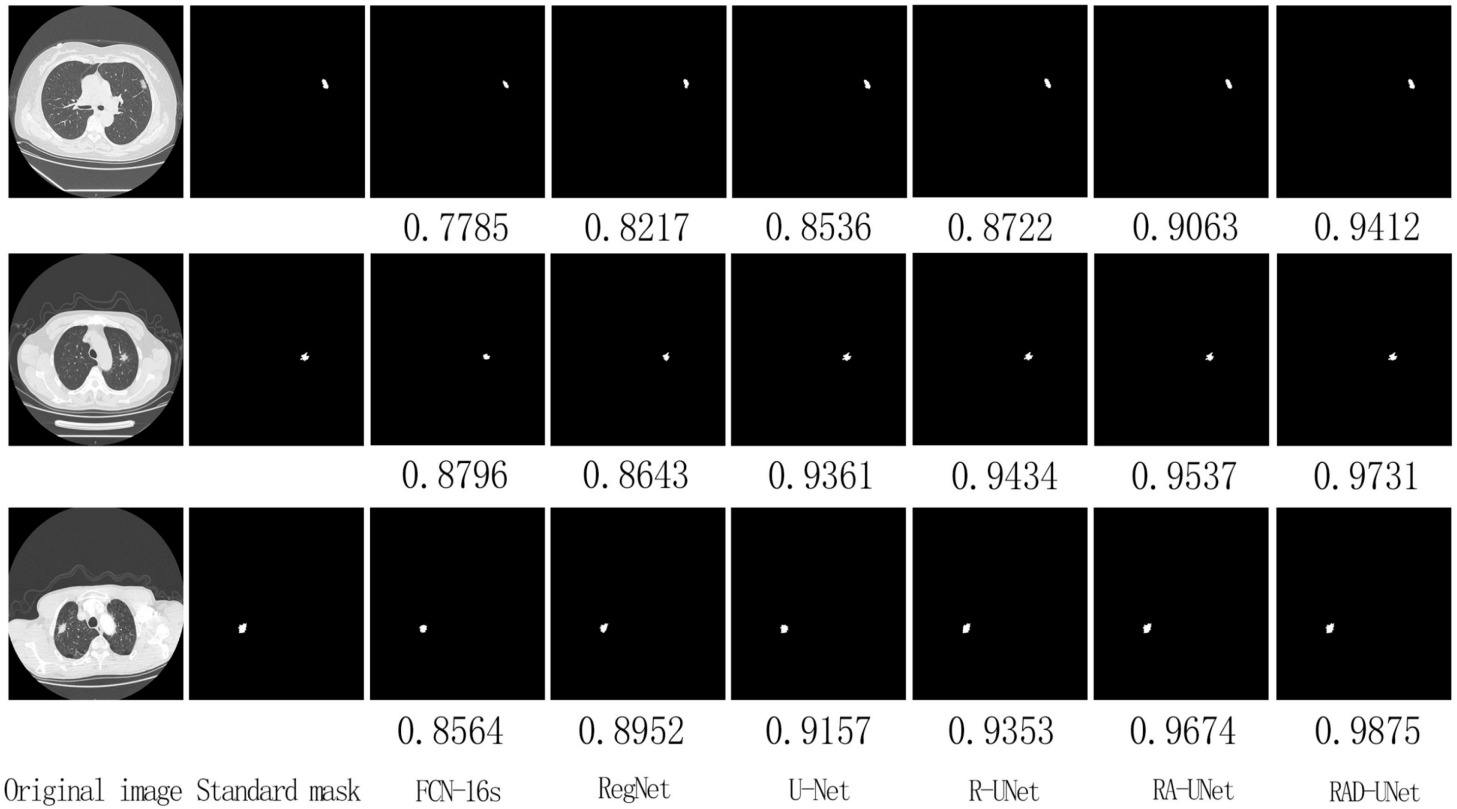
Comparison of lung nodule segmentation results on the AHAMU-LC dataset.

On the LIDC dataset, the improved RAD-UNet model was compared with R-UNet, RA-UNet, SegNet (14) and U-Net (15). The segmentation algorithms were compared and evaluated. The experimental results are shown in **Figure 7**, including the original CT images in the test set, the lesions labelled by a doctor, and the lung nodules segmented by the SegNet (14), U-Net (15), R-UNet, RA-UNet, and RAD-UNet models.

**Figure 7** shows that the RAD-UNet algorithm proposed in this paper produces comparable segmented lung CT images. The nodules with small, blurry edges and similar background grey values identified by RAD-UNet are significantly better than the nodules identified by SegNet (14) and U-Net. SegNet (14) and U-Net (15) have different degrees of over- and undersegmentation at the boundary.

**Figure 8:**
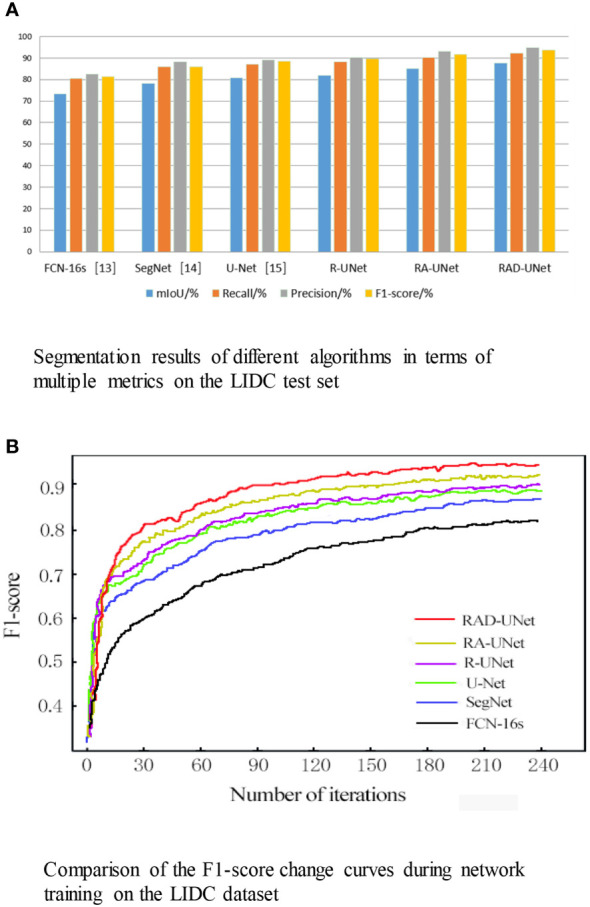
**(A)** Segmentation results of different algorithms in terms of multiple metrics on the LIDC test set. **(B)** Comparison of the F1-score change curves during network training on the LIDC dataset.

The segmentation experiment results on the LIDC dataset are shown in **Table 1** and **Figure 8A**. As shown in **Table 1** and **Figure 8A**, the method proposed in this paper performs better than the SegNet (14) and U-Net (15) algorithms on all evaluation metrics; on the test set, the mIoU of the proposed model reached 87.76%, the recall reached 92.17%, the precision reached 94.75%, and the F1-score reached 93.56%.

**Table 1:** Segmentation results of different algorithms in terms of multiple metrics on the LIDC test set.

Algorithm	mIoU/%	Recall/%	Precision/%	F1-score/%
FCN-16s[13]	73.21	80.63	82.52	81.35
SegNet[14]	78.13	85.98	88.13	86.08
U-Net[15]	80.72	87.05	89.12	88.67
R-UNet	81.93	88.13	90.33	89.79
RA-UNet	85.15	90.21	93.06	91.63
RAD-UNet	87.76	92.17	94.75	93.56

To visualize the performance of the segmentation model, the F1-scores for RAD-UNet, RA-UNet, R-UNet, SegNet (14), and U-Net (15) during training are plotted in **Figure 8B**.

**Figure 8B** shows that SegNet (14) and U-Net (15) obtained similar results, while RAD-UNet performs better than these two models. The parameter adjustment in the pyramid pooling module, as well as the cross fusion of the global and local semantic features, improve the F1-score of the proposed model after convergence in the training process. Thus, RAD-UNet achieves more accurate and fine segmentation of small target lung lesions in the CT images than the SegNet (14) and U-Net (15) algorithms.

### 5.3 Experimental results on the AHAMU-LC dataset

**Figure 9:**
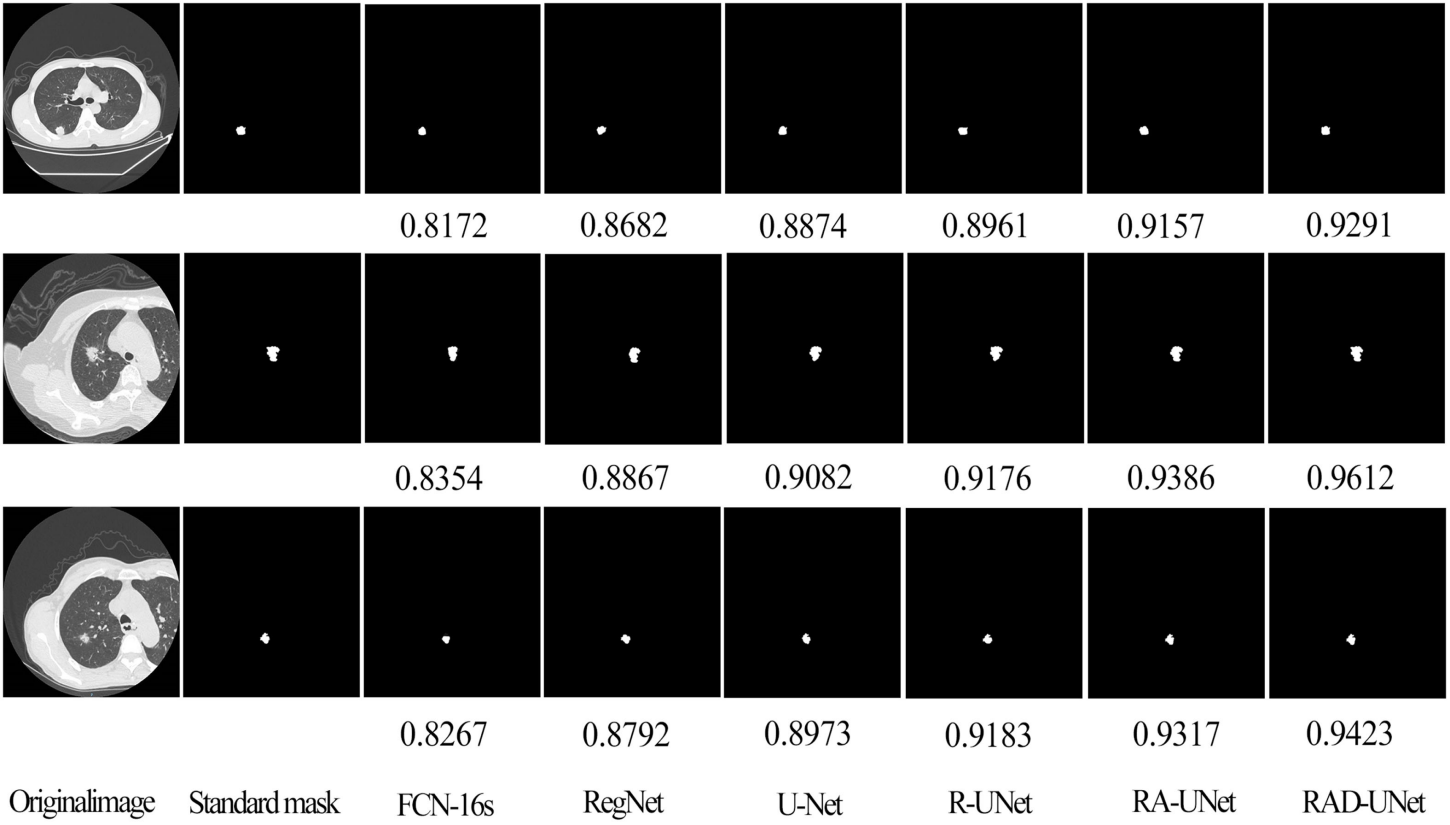
Comparison of lung nodule segmentation results on the AHAMU-LC dataset.

To further demonstrate the robustness of the improved RAD-UNet algorithm, comparative experiments were also performed on the AHAMU-LC dataset. **Figure 9** shows the RAD-UNet, R-UNet, RA-UNet, SegNet (14), and U-Net (15) experimental results on this dataset. When the nodule infiltrates the surrounding environment, the SegNet (14) and U-Net (15) algorithms extract information from different semantic-level features due to blurring and boundary inadequacy; thus, the invading tumour cannot be accurately identified, and undersegmentation occurs. The manual segmentation of the results by doctors as a standard mask is more consistent with the results in this article.

**Figure 10:**
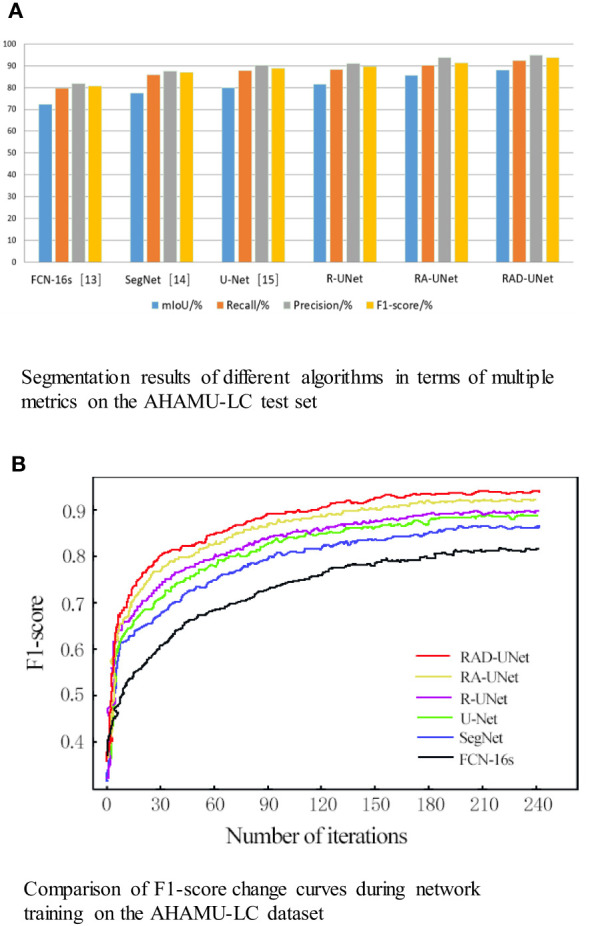
**(A)** Segmentation results of different algorithms in terms of multiple metrics on the AHAMU-LC test set. **(B)** Comparison of F1-score change curves during network training on the AHAMU-LC dataset.

To quantitatively analyse and verify the effectiveness of the improved RAD-UNet algorithm, the RAD-UNet, SegNet (14) and U-Net (15) methods were applied to the AHAMU-LC dataset. Comparative experiments were performed on the test set of the dataset, and the results are shown in **Table 2** and **Figure 10A**. **Table 2** and **Figure 10A** shows that the method proposed in this paper achieved better evaluation metric scores than the other methods, and its mIoU on the test set reached 88.13%, the recall reached 92.32%, the precision reached 94.82%, and the F1-score reached 93.72%.

**Table 2:** Segmentation results of different algorithms in terms of multiple metrics on the AHAMU-LC test set.

Algorithm	mIoU/%	Recall/%	Precision/%	F1-score/%
FCN-16s[13]	72.24	80.23	82.31	81.62
SegNet[14]	77.57	85.86	87.59	86.87
U-Net[15]	79.86	87.63	90.14	88.73
R-UNet	81.64	88.36	91.07	89.71
RA-UNet	85.73	90.24	93.62	91.25
RAD-UNet	88.13	92.32	94.82	93.72

To visualize the performance of the improved RAD-UNet segmentation model, the accuracy curve of each comparison algorithm during training is plotted, as shown in **Figure 10B**.

**Figure 10B** demonstrates that by training the model on a large number of images, the proposed method can extract multilevel abstract features. Moreover, after convergence, the RAD-UNet algorithm is more accurate than the SegNet (14) and U-Net (15) models, resulting in a more accurate segmentation of small lung lesions in the CT images and a more robust model.

### 5.4 Discussion of experimental results

The proportion of pixels in the entire CT image is small for lung nodule lesions, and it is difficult to extract small target features and train the network. Additionally, the similarity between lung nodule lesions and normal tissues of CT images is high. It is difficult to distinguish them from background images and to extract strong distinguishable features. In this experiment, the U-Net network was selected as the baseline method, and the above lung nodule image segmentation problems were targeted on the basis of the U-shaped encoding-decoding network. The U-Net network was studied, improved and optimized to solve the difficulty of image segmentation of lung nodules.

It can be seen from the above test results that the three improved parts, R-UNet, RA-UNet and RAD-UNet, improved their performance to a certain extent compared with SegNet (14) and UNet. Their working principles and contributions are discussed as follows:

R-UNet uses residual blocks to replace the coder of the UNet network for improvement. Because the residual blocks in the residual network use the jump connection mode, in the depth neural network, the gradient disappears because the increase in depth is reduced. Compared with UNet, it not only improves the segmentation accuracy but also reduces the training time and the number of parameters.

It can be seen in **Table 1** and **Table 2** that R-UNet is better than UNet in the four evaluation indicators of mIoU, recall, precision and F1-score, which are 1.21% 1.78%, 1.08% 0.73%, 0.21% 0.93% and 1.12% 0.98% higher, respectively. This proves the effectiveness of replacing the UNet network encoder with a residual structure block.

Inspired by the structural idea of ASPP modules proposed by Chen et al. in DeepLab networks. The ASPP module has a good effect on extracting the multiscale features of the image. The smaller the void rate of the convolution kernel of the ASPP module is, the more conducive it is to segmenting smaller targets, and the larger the void rate of the convolution kernel is, the more conducive it is to segmenting larger targets.

In this experiment, in view of the small characteristics of lung imaging lesions, after the U-Net (15) coding structure, the modified ASPP was added to improve the latter ASPP module. The latter ASPP module consists of a 1×1 convolution and a 3×3 convolution with a void ratio of 2, a 3×3 convolution with a void rate of 4, and a 3×3 convolution with a void rate of 6 convolutions and an average pooling composition. Convolution kernels with void ratios of 2, 4, and 6 are used to increase the ability of the neural network to segment smaller targets of lung imaging lesions.

RA-UNet uses increased cavity convolution to fuse feature maps extracted with different cavity rates, expand the receptive field, enhance feature expression, and improve the feature extraction ability and segmentation effect for small lung lesions.

It can be seen in **Table 1** and **Table 2** that RA-UNet is superior to R-UNet in the four evaluation indicators of mIoU, recall, precision and F1 core, which are 3.22% 3.09%, 2.08% 1.88%, 2.73% 2.55% and 1.84% 1.54% higher, respectively. This proves the advantage of adding cavity convolution.

In view of the problem that lung nodule lesions are not clearly distinguished in CT images, a DFCF module with feature cross-fusion was added after each upsampling in the decoder of the U-Net baseline network. DFCF makes the effective feature weight larger, the invalid or small effect feature weight smaller, enhances the ability to distinguish between lung nodules and background, and improves the channel attention module in DFCF to CBAM attention module with both channel attention and spatial attention. The network model considers both the importance of pixels at different locations of the same channel and the importance of pixels in different channels.

RAD-UNet uses a nonlocal attention mechanism to cross-fuse global and local semantic features, integrate important features at different levels, enhance the network’s ability to distinguish between lung lesions and normal tissues, further improve the segmentation performance, and prove that the improved feature fusion module has also made some contributions.

It can be seen in **Table 1** and **Table 2** that RAD-UNet is better than RA-UNet in the four evaluation indicators of mIoU, recall, precision and F1-score, which are 2.61% 2.40%, 1.91% 2.08%, 1.69% 1.20% and 1.93% 2.47% higher, respectively. It shows the effectiveness and superiority of adding cross-fusion global and local semantic features.

### 5.5 Discussion of classification test results

To further test the classification results of lung nodule segmentation, CT images of lung nodules of different diameters and different numbers were randomly selected from the above dataset, and the trained improved RAD-UNet model and the classical network model U-Net (15) were used for classification testing. The results are shown in **Table 3**.

**Table 3:** Classification test segmentation results of lung nodules of different sizes using U-Net (15) and RAD-UNet.

			U-Net (15)				RAD-UNet			
Cate	Diameter(mm)	Amount	mIoU/%	Recall/%	Precision/%	F1-score/%	mIoU/%	Recall/%	Precision/%	F1-score/%
Micro nodule	d ≤ 5	437	68.56	78.64	85.34	84.21	80.37	86.74	90.13	91.57
Small nodule	5<d ≤ 10	816	72.63	81.53	87.67	85.37	82.72	90.53	92.56	92.66
Nodule	10<d ≤ 30	573	76.12	85.61	89.71	87.55	86.55	91.82	94.23	93.12
Lung mass	d>30	85	84.36	88.39	91.13	90.03	89.17	93.65	95.21	93.57

It can be seen in **Table 3** of the classification test that with the decrease in lung nodule diameter, the improved RAD-UNet has obvious advantages over U-Net (15) network segmentation in the four evaluation indicators of mIoU, recall, precision and F1-score. It was further confirmed that the improved model RAD-UNet had a good segmentation effect on pulmonary nodule lesions with a small proportion of target pixels in CT images and was very similar to the environment.

### 5.6 Quantitative evaluation with statistical analysis

To quantitatively evaluate, we compare the effectiveness of the proposed method RAD-UNet with the deep learning models U-Net (15) and SegNet (14) segmented with CT lung nodule images from the above dataset, as shown in **Table 4**. The proposed method exceeds the baseline technique in the segmentation of lung nodule images, with an mIoU of 87.8%, a recall of 92.2%, an accuracy of 94.8%, and an F1 score of 93.6%.

**Table 4:** Quantitative evaluation of the proposed method and the baseline approaches in the segmentation of CT images of lung nodules.

Method		Score			95% C.I.for Mean	
		Mean	Std.Deviation	Std.Error	Lower Bound	Upper Bound
mIoU	Proposed method	**0.878**	0.104	0.048	0.746	0.924
	U-Net[15]	0.807	0.103	0.072	0.676	0.915
	SegNet[14]	0.781	0.116	0.061	0.679	0.823
	FCN-16S[13]	0.732	0.143	0.087	0.612	0.864
Recall	Proposed method	**0.922**	0.034	0.042	0.813	0.962
	U-Net[15]	0.871	0.021	0.037	0.758	0.957
	SegNet[14]	0.860	0.026	0.029	0.754	0.963
	FCN-16S[13]	0.806	0.153	0.082	0.701	0.952
Precision	Proposed method	**0.948**	0.128	0.039	0.832	0.997
	U-Net[15]	0.891	0.105	0.028	0.779	0.976
	SegNet[14]	0.881	0.087	0.023	0.768	0.984
	FCN-16S[13]	0.825	0.153	0.061	0.703	0.946
F1-score	Proposed method	**0.936**	0.119	0.030	0.827	0.991
	U-Net[15]	0.887	0.085	0.022	0.782	0.975
	SegNet[14]	0.861	0.116	0.031	0.753	0.981
	FCN-16S[13]	0.814	0.127	0.056	0.711	0.947

Bold values is the highest value of several methods of the same indicator.

To further demonstrate the efficacy of the proposed method, using SPSS software, we examined the quantitative scores that were evaluated with Fisher’s least significant difference (LSD) procedure (**Table 5**). Based on the LSD test, the suggested approach exceeds the baseline approaches in terms of mIoU, recall, precision and F1-score (p< 0.001).

**Table 5:** Multiple comparisons of CT lung nodule segmentation results: LSD test.

LSD Multiple Comparisons						
Dependent Variable	(I) Method	(J) Method	Mean Difference(I-J)	Sig.	95% C.I.	
					Lower Bound	Upper Bound
mIoU	Proposed method	U-Net[15]	0.071*	<0.001	0.062	0.093
		SegNet[14]	0.097*	<0.001	0.085	0.127
		FCN-16s[13]	0.146*	<0.001	0.126	0.167
Recall	Proposed method	U-Net[15]	0.051*	<0.001	0.039	0.075
		SegNet[14]	0.062*	<0.001	0.051	0.078
		FCN-16s[13]	0.116*	<0.001	0.102	0.134
Precision	Proposed method	U-Net[15]	0.057*	<0.001	0.048	0.077
		SegNet[14]	0.067*	<0.001	0.054	0.086
		FCN-16s[13]	0.123*	<0.001	0.107	0.142
F1-score	Proposed method	U-Net[15]	0.049*	<0.001	0.037	0.063
		SegNet[14]	0.075*	<0.001	0.062	0.091
		FCN-16s[13]	0.122*	<0.001	0.113	0.137

* The proposed method is significantly better than the baseline approaches using the LSD test (p< 0.001).

## 6 Conclusion

SegNet (14) and U-Net (15) obtain undersegmented results when extracting lung lesions in CT images due to the small target size and insignificant discrimination from the background. In this paper, an improved RAD-UNet neural network model is proposed, which replaces the U-Net (15) convolutional network encoder with a residual network module and introduces a pyramid pooling module with optimized parameters, cross-fusion semantic features and other improvements, thereby enabling end-to-end, pixel-to-pixel processing in the convolutional network. The experimental results on the LIDC and AHAMU-LC datasets show that compared to the conventional SegNet (14) and U-Net (15) segmentation networks, RAD-UNet’s mIoU reached 87.76% and 88.13% on the two datasets, and the F1-score reached 93.56% and 93.72%, respectively. The results objectively illustrate that the proposed RAD-UNet algorithm segments lung nodules more accurately than the conventional SegNet (14) and U-Net models in lung CT images.

## Data availability statement

Publicly available datasets were analyzed in this study. This data can be found here: https://wiki.cancerimagingarchive.net/display/Public/LIDC-IDRI.

## Author contributions

ZW conceived and experimented with the effect of deep learning improvement algorithm on CT image semantic segmentation. JX tested the improved algorithm. XL and JZ provide, sift, and confirm manual segmentation controls for lung CT images. All authors contributed to the article and approved the submitted version.
